# Acute Effects of Electronic and Tobacco Cigarette Smoking on Sympathetic Nerve Activity and Blood Pressure in Humans

**DOI:** 10.3390/ijerph19063237

**Published:** 2022-03-09

**Authors:** Kyriakos Dimitriadis, Krzysztof Narkiewicz, Ioannis Leontsinis, Dimitris Konstantinidis, Costas Mihas, Ioannis Andrikou, Costas Thomopoulos, Dimitrios Tousoulis, Konstantinos Tsioufis

**Affiliations:** 1 First Cardiology Clinic, Medical School, National and Kapodistrian University of Athens, Hippokration Hospital, 11527 Athens, Greece; dimitriadiskyr@yahoo.gr (K.D.); giannisleontsinis@gmail.com (I.L.); dim_konstantinidis@hotmail.com (D.K.); gas521@yahoo.co.uk (C.M.); ioannis_andrikou@yahoo.gr (I.A.); thokos@otenet.gr (C.T.); drtousoulis@med.uoa.gr (D.T.); 2 Department of Hypertension and Diabetology, Medical University of Gdansk, 80-210 Gdansk, Poland; krzysztof.narkiewicz@gumed.edu.pl

**Keywords:** blood pressure, electronic cigarette, heart rate, sympathetic nervous system, tobacco cigarette

## Abstract

Acute tobacco cigarette (TC) smoking increases blood pressure and sympathetic nerve activity, whereas there are scarce data on the impact of electronic cigarette (EC) smoking. We assessed the acute effects of TC, EC and sham smoking on blood pressure, heart rate and sympathetic nervous system. **Methods:** We studied 12 normotensive male habitual smokers (mean age 33 years) free of cardiovascular disease. The study design was randomized and sham controlled with three experimental sessions (sham smoking, TC smoking and EC smoking). After baseline measurements at rest, the subjects were then asked to smoke (puffing habits left uncontrolled) two TC cigarettes containing 1.1 mg nicotine, EC smoking or simulated smoking with a drinking straw with a filter (sham smoking), in line with previous methodology. **Results:** EC smoking at 5 and 30 min compared to baseline was accompanied by the augmentation of mean arterial pressure (MAP) and heart rate (*p* < 0.001 for all). The muscle sympathetic nerve activity (MSNA) decrease was significant during both TC and EC sessions (*p* < 0.001 for both comparisons) and was similar between them (−25.1% ± 9.8% vs. −34.4% ± 8.3%, respectively, *p* = 0.018). Both MSNA decreases were significantly higher (*p* < 0.001 for both comparisons) than that elicited by sham smoking (−4.4% ± 4.8%). Skin sympathetic nerve activity increase was significant in both TC and EC groups (*p* < 0.001 for both comparisons) and similar between them (73.4% ± 17.9% and 71.9% ± 7%, respectively, *p* = 0.829). **Conclusions:** The unfavorable responses of sympathetic and arterial pressure to EC smoking are similar to those elicited by TC in healthy habitual smokers.

## 1. Introduction

Smoking constitutes one of the major preventable causes of cardiovascular disease worldwide [1,2,3]. Although smoking rates have been importantly reduced during the last decades, there is still a persistence to this unhealthy habit that is coupled with the diversification of it in the form of electronic cigarettes (ECs) [1,4]. The upsurge in ECs is a worrying current global trend since there is a lack of longitudinal data on their safety and health effects, but, yet, they are proposed as less harmful alternatives [1,4,5,6,7]. 

Understanding the pathophysiological links of smoking to adverse cardiovascular outcome was fundamental towards implementing health policies of smoking cessation [1,2,3]. The detrimental impact of tobacco cigarette (TC) smoking on hemodynamics, endothelium, mechanisms of atherosclerosis, thrombosis, diffuse inflammation and oxidative stress have been thoroughly examined [4,5,6]. On the other hand, the data on vaping are currently building up showing adverse systemic effects similar to TC but mostly in the acute phase [1,7,8,9,10,11]. Focusing on the impact on autonomic function, TC is related to impaired heart rate variability [12,13] and unfavorable changes in muscle sympathetic nerve activity (MSNA) and skin sympathetic nerve activity (SSNA) [4,14,15,16,17,18,19]. However, the link of EC consumption with sympathetic modulation has not been adequately explored with most evidence coming from studies assessing the changes in heart rate variability [1,20,21,22,23]. There is only one recent study showing the link of vaping with acute increases in arterial pressure and unfavorable autonomic regulation in healthy non-smokers [23].

Based on the above, to explore the potential mechanisms of the adverse effects of EC smoking on the cardiovascular and autonomic system, it would be helpful to compare the impact of EC and TC smoking on sympathetic activity by the means of microneurography and on hemodynamic parameters. Thus, we assessed the acute effects of TC and EC exposures on MSNA and SSNA as well as on arterial pressure in healthy habitual TC smokers. 

## 2. Material and Methods

### 2.1. Subjects

Healthy male volunteers between the ages of 25 and 45 years were eligible for enrollment if they met the following criteria: reference [1] no current (within 1-year) EC smoking, [2] body mass index ≤30 kg/m^2^; reference [3] no known health problems; reference [4] alcoholic intake ≤2 drinks per day and no illicit drug use (determined through screening questionnaire); reference [5] lack of exposure to secondhand smoke, or using licensed nicotine replacement therapies; reference [6] current TC smoking (>10 cigarettes daily). 

A total of 15 participants meeting the above criteria were enrolled in this study. Three subjects completed only part of the protocol procedures and were excluded. Thus, we evaluated the effects of smoking in 12 healthy normotensive habitual male TC smokers (mean age: 34 ± 4 years and body mass index: 24 ± 3 kg/m^2^), free of diabetes mellitus, hyperlipidemia, or any other systemic disease, after detailed clinic and laboratory examinations, who were taking no medication. 

### 2.2. Measurements

Subjects abstained from caffeine, ethanol and nicotine for at least 12 h before each session and were tested in the supine position in a temperature-controlled (23 °C) room. After instrumentation, the subjects were asked to remain quiet for an acclimation period of 5 min or until the hemodynamic variables and MSNA and SSNA recordings were stable. Heart rate was measured continuously by an ECG, and blood pressure was measured beat-to-beat by an automatic plethysmograph (Finometer MIDI, Finapres Medical Systems BV, Institutenweg 25, 7521 PH Enschede The Netherlands). In more detail, MSNA was obtained through a tungsten microelectrode inserted into the right or left peroneal nerve posterior to the fibular head. The microelectrode is made of tungsten with a diameter of 200 mm in the shaft, tapering to an uninsulated tip of 1–5 mm. A reference electrode is positioned subcutaneously 1–3 cm from the recording electrode and serves as a ground. The nerve signal was amplified according to prespecified algorithms, fed through a bandpass filter (700–2000 Hz) and integrated with a nerve traffic analyzer (ML 185 NeuroAmpEx; ADInstruments Ltd., Oxford, UK). Integrated nerve activity was monitored and the MSNA and SSNA were assessed according to established methodology [24,25,26]. Neurograms were accepted only if they did not show simultaneous skin and muscle sympathetic nerve traffic and if the signal-to-noise ratio was more than 3 [3,8,9]. The MSNA and SSNA were identified by their characteristic morphology and relationship to the R waves on the ECG and quantified as bursts’ frequency (bursts per minute). The measurement has been shown to be highly reproducible with an intra-observer reproducibility of around 3.8–4.5% [24,25,26].

Plasma norepinephrine levels were measured by high-performance liquid chromatography with electrochemical detection as previously reported. Inter-assay and intra-assay coefficients are 3.86% and 3.64%, respectively. Blood samples were obtained at baseline and after completion of each phase of TC and EC smoking sessions. No blood samples were collected for sham smoking.

### 2.3. Experimental Session

**Figure 1:**
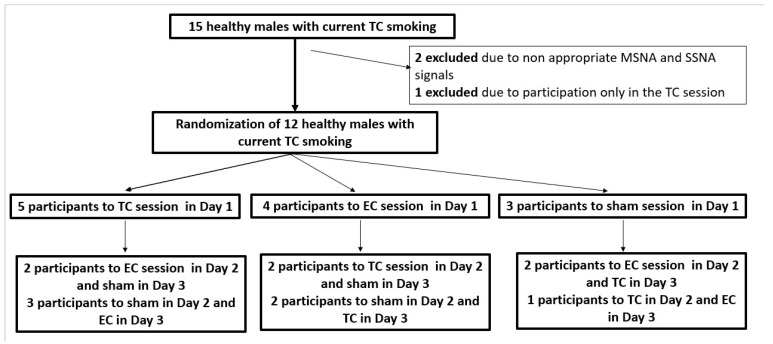
The study design illustrating the randomization to TC, EC and sham smoking sessions in random order, each session on a separate day. EC, electronic cigarette; MSNA, muscle sympathetic nerve activity; SSNA, skin sympathetic nerve activity; TC, tobacco cigarette.

The study design was randomized and sham controlled with 3 experimental sessions (sham smoking, TC smoking and EC smoking). The sessions were performed in random order, each session on a separate day. Randomization was performed with means of closed envelopes for each subject and session (Figure 1). After baseline measurements at rest, the subjects were then asked to smoke (puffing habits left uncontrolled) 2 TC cigarettes containing 1.1 mg nicotine, smoke an EC or simulate smoking with a drinking straw with a filter (sham smoking), in line with previous methodology [16,17,27]. The first TC cigarette or sham smoking (Phase 1 of TC or sham smoking) was separated by 5 min from the second TC cigarette or sham smoking (Phase 2 of TC or sham smoking). Regarding EC smoking, participants were advised every 30 s to place the EC in mouth, inhale for 3 s, hold aerosol in for 3 s and then exhale. The time points for measurements in EC smoking were the period of 5 min (Phase 1) and 30 min (Phase 2) as previously suggested [20,21,22]. The EC used in the study was one with tobacco-flavored liquid, vegetable glycerin/propylene glycol solvents with 1.2% nicotine. Changes in MSNA, SSNA, blood pressure and heart rate were calculated for the last 2 min of each smoking phase, when the effects of smoking are especially marked [15,16]. The present study complies with the Declaration of Helsinki and the locally appointed ethics committee has approved the research protocol. Informed consent has been obtained from all subjects.

### 2.4. Statistical Analysis 

Sample size was based on end points of MSNA and SSNA. Because there were no data regarding the acute effects of EC on sympathetic nervous system components, we used the reported pooled SD of MSNA and SSNA after TC smoking [16,17]. Assuming similar SDs with EC use, a sample size of 10 subjects was required in order to achieve a statistical power of 80% power with a two-sided alpha = 0.05. Our final analysis included 12 subjects. Data were analyzed by repeated-measures analysis of variance (ANOVA) using Box’s conservative epsilon, considering time (before versus during smoking) as the within factor and session (sham smoking, TC smoking alone and EC smoking) as the between factor. The *p*-values for differences within a session were obtained by post hoc tests (planned contrasts). Results are expressed as mean ± standard deviation (SD). All tests were two-sided and *p* < 0.002 was considered significant after Bonferroni adjustment for multiple comparisons. STATA^®^ v.16.0 (StataCorp, College Station, TX 77845, USA) statistical software was used for the analysis. 

## 3. Results

At the start of the TC, EC and sham smoking sessions there were no differences regarding baseline MAP, heart rate, MSNA and SSNA (*p* = NS for all, Table 1 and Table 2).

### 3.1. Effects of Smoking on Hemodynamic Parameters

**Figure 2:**
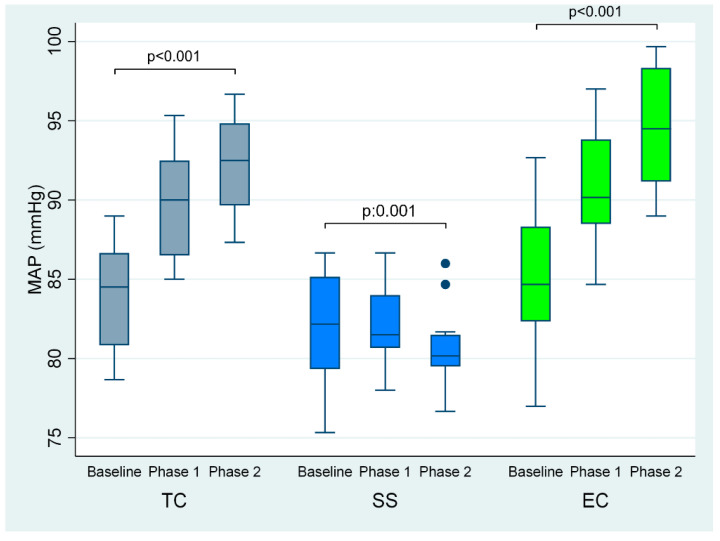
MAP at baseline and phases 1 and 2 of TC, EC and sham smoking. EC, electronic cigarette; MAP, mean arterial pressure; TC, tobacco cigarette, SS, sham smoking.

After the first and second phases of tobacco cigarette smoking, there was a significant increase in mean arterial pressure (by 6 and 8 mmHg, respectively, overall *p* < 0.001) and heart rate (by 8 and 12 beats/minute, respectively, overall *p* < 0.001) (Table 1, Figure 2), compared to baseline. Similarly, EC smoking at 5 and 30 min compared to baseline was accompanied by augmentation in mean arterial pressure (by 6 and 10 mmHg, respectively, overall *p* < 0.001) and heart rate (by 5 and 9 beats/minute, respectively, overall *p* < 0.001) (Table 1, Figure 2). Regarding sham smoking, it was accompanied by a reduction in mean arterial pressure (by 2 and 4 mmHg, respectively, *p* = 0.001) after the first and second cigarette compared to baseline, whereas there was no significant difference in heart rate (*p* = 0.041) (Table 2, Figure 2). 

### 3.2. Effects of Smoking on Sympathetic Nervous System 

**Figure 3:**
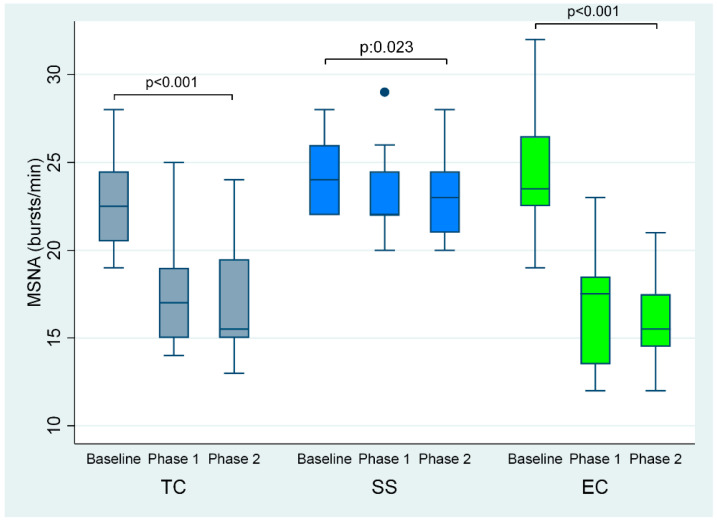
MSNA at baseline and phases 1 and 2 of TC, EC and sham smoking. EC, electronic cigarette; MSNA, muscle sympathetic nerve activity; TC, tobacco cigarette, SS, sham smoking.

**Figure 4:**
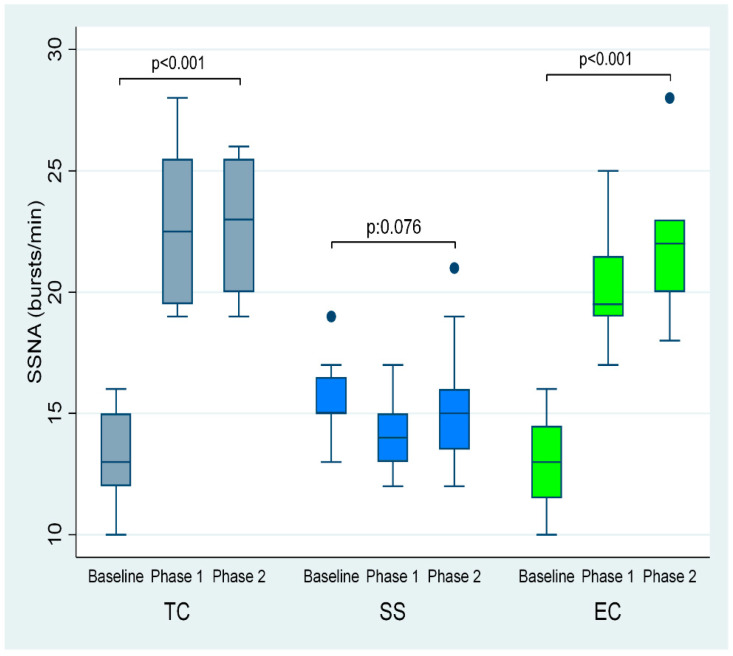
SSNA at baseline and phases 1 and 2 EC and sham smoking. EC, electronic cigarette; SSNA, skin sympathetic nerve activity; TC, tobacco cigarette, SS, sham smoking.

The first and second TC smoking phases were accompanied by lower MSNA (by 6 and 6 bursts/minute, respectively, overall *p* < 0.001) compared to baseline, whereas SSNA was increased (by 9 and 10 bursts/minute respectively, overall *p* < 0.001) (Table 1, Figure 3 and Figure 4).

Additionally, EC smoking at 5 and 30 min compared to baseline caused a decrease in MSNA (by 8 and 8 bursts/minute, respectively, overall *p* < 0.001) and an augmentation in SSNA (by 7 and 9 bursts per minute, respectively, overall *p* < 0.001) (Table 1, and Figure 3 and Figure 4). Plasma norepinephrine levels were significantly increased after TC but not after EC smoking (Table 1). 

Sham smoking had no significant effect on MSNA (*p* = 0.023) and SSNA (*p* = 0.076) (Table 2 and Figure 3 and Figure 4].

**Figure 5:**
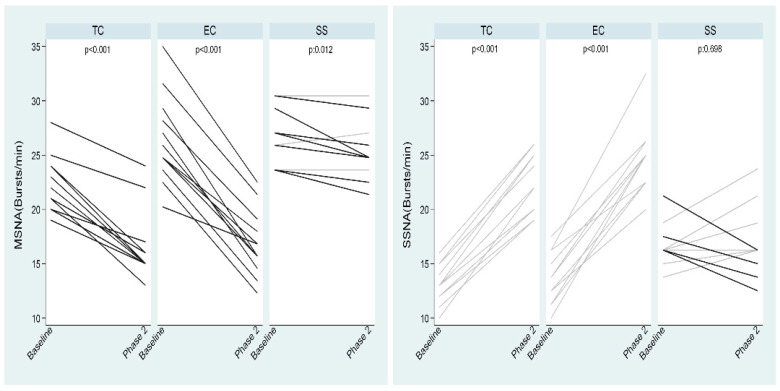
Changes in MSNA and SSNA by TC, EC and sham smoking. EC, electronic cigarette; MSNA, muscle sympathetic nerve activity; SSNA, skin sympathetic nerve activity; TC, tobacco cigarette, SS, sham smoking.

The MSNA decrease was significant during both TC and EC sessions (*p* < 0.001 for both comparisons) and was similar between them (−25.1% ± 9.8% vs. −34.4% ± 8.3%, respectively, *p* = 0.018). Both decreases were significantly higher (*p* < 0.001 for both comparisons) than that elicited by sham smoking (−4.4% ± 4.8%), although this was not significant by itself (*p* = 0.012) (Figure 5). SSNA increase was significant in both TC and EC groups (*p* < 0.001 for both comparisons) and similar between them (73.4% ± 17.9% and 71.9% ± 7%, respectively, *p* = 0.829). In addition, this difference was significantly higher (*p* < 0.001 for both comparisons) than that during sham smoking where a slight but not statistically significant mean reduction was found (−1.3% ± 17.9%, *p* = 0.698) (Figure 5).

There was no association of the change in MAP with the alterations in MSNA and SSNA during sessions of TC, EC and sham smoking (data not shown). Moreover, norepinephrine levels were not related to MSNA and SSNA changes (data not shown). 

## 4. Discussion

**Figure 6:**
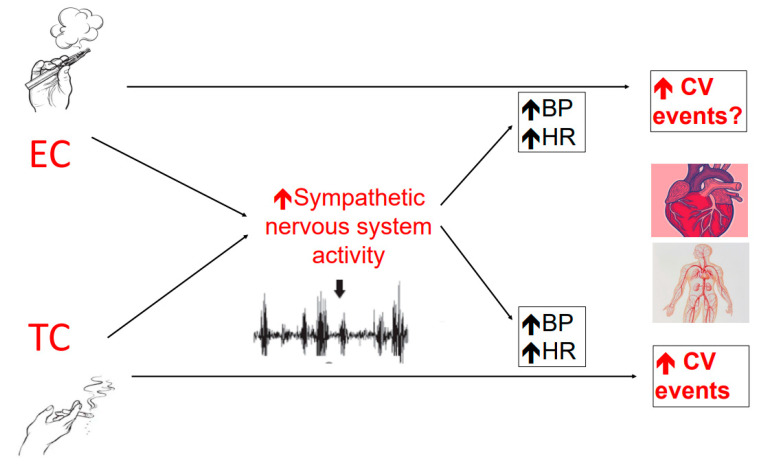
EC smoking, similar to TC smoking, may act at a central level to cause a uniform increase in sympathetic nerve traffic to blood vessels, skin and the heart. Alterations in sympathetic drive are paralleled by an increase in blood pressure and heart rate, reflecting a systemic hemodynamic response to smoking of both types of cigarettes. These unfavorable effects of ECs mimicking those by TCs may chronically lead to increased risk of cardiovascular events. BP, blood pressure; CV, cardiovascular; EC, electronic cigarette; HR, heart rate; TC, tobacco cigarette.

To the best of our knowledge, this is the first study to have assessed the effect of both TC and EC smoking on MSNA and SSNA in healthy smokers. Our findings indicate that ECs have a powerful sympathetic excitatory effect, similar to that elicited by TCs. EC smoking may act at a central level to cause a uniform increase in sympathetic nerve traffic to blood vessels, skin and the heart. Alterations in sympathetic drive are paralleled by an increase in blood pressure and heart rate, reflecting a systemic hemodynamic response to smoking both types of cigarettes (Figure 6). 

Previous studies have shown that TC smoking causes a marked elevation in blood pressure by sympathetic excitation, impairment of arterial baroreflex function and acute reduction in arterial compliance [15,16,17,18,19,28]. The effect of TC on sympathetic drive is complex [16]. TC causes a marked increase in skin sympathetic discharge and a decrease in MSNA. Paradoxical MSNA decrease might at first glance suggest a sympathoinhibitory effect of TC. However, MSNA, in contrast to SSNA, is inhibited by blood pressure changes [15,16,17]. Therefore, MSNA inhibition during TC smoking reflects the protective effect of arterial baroreflexes, responding to increases in blood pressure. When the blood pressure increase in response to TC smoking is blunted by the simultaneous infusion of sodium nitroprusside, there is a striking increase in MSNA [16]. 

The recently published study by Gonzalez J et al. [23] in healthy non-smokers, describe similar results concerning the effect of ECs on MSNA and arterial pressure. However, there is no estimation of SSNA [23] that is not influenced by hemodynamic load changes due to smoking. Thus, our study provides for the first time, evidence that EC increases SSNA in humans. The rise in SSNA was evident from the first TC and the start of EC smoking and remained high till the end of the sessions. Although we did not seek to maintain pressure at baseline levels by sodium nitroprusside, as performed in a previous study [16], our current findings indicate that the effects of TC and EC smoking on MSNA are comparable. The sympathoexcitatory effect of ECs, in addition to SSNA increase, is further supported by a significant rise in plasma norepinephrine and tachycardia, which were of a similar magnitude to those during the TC session. Importantly, blood pressure increase was similar during TC and EC sessions, facilitating interpretation of the sympathetic traffic results. This is in line with a previous work [23], highlighting the unfavorable impact of vaping on arterial pressure. The use of the control arm strengthens our findings since sham smoking was associated with a lack of significant MSNA change and a significantly lower increase in SSNA compared to TC and EC smoking.

The acute tachycardic effect of TC smoking had a similar pattern to that observed during the EC session. Although it is established that nicotine, as a sympathomimetic substance, in TCs induce the increase in cardiac sympathetic nerve activity reflected by increased heart rate, the contribution of other non-nicotine combusted organic constituents is largely unexplored [4,15,16,17,18,19,20,21,22,27]. Especially in the EC setting, the data are scarce, supporting mostly the main contribution of nicotine to EC-related unfavorable change in cardiac sympathetic nerve activity as observed in heart rate variability parameters as well as blood pressure increase [1,20,22,23,26]. Adding more fuel to this controversy, air pollution with similarities to TC smoke without nicotine can increase cardiac sympathetic drive via reactive oxygen species and pathways of lung vagal afferent C-fibers [22,28,29,30]. Nowadays, there are data that show that, although particulate size and number in ECs have considerable overlap with air pollution and TC smoke, the levels of toxic substances are lower. In addition, it is supported that flavorings, solvents and/or contaminants in ECs are not linked to acute sympathetic system changes [22,30], as evident in vaping without nicotine [23].

Norepinephrine levels that are not specific to MSNA and SSNA and reflect acute changes in nervous tone, were found to be increased at the end of TC but not EC sessions compared to baseline, and, also, they were not related to the parameters of microneurography. This could be due to both MSNA and SSNA being influenced by nicotine and diverse axes and feedback mechanisms, while norepinephrine level alterations are the severe result of the nicotine-induced local release of catecholamines from adrenergic nerve terminals [4]. Moreover, given that the studied EC contained nicotine, one could not exclude different reactions of non-nicotine ECs, as explored recently [23]. From a pathophysiological point of view, nicotine and other toxic agents such as the gases and fine particulate matter (PM2.5; defined as <2.5 mm in hydrodynamic diameter) in cigarette smoke are implicated in the pressor result and autonomic system shifting to sympathetic predominance [1,4]. Adjunct mechanisms for the observed smoking effects include a 1-adrenoceptor–mediated vasoconstriction, vasopressin release and endothelial dysfunction [1,4,31,32]. 

The subjects in the current study were males and healthy with preserved baroreflex axis. This is not the case for patients with hypertension, diabetes mellitus and heart failure who are characterized by baroreflex failure and might be more sensitive to TC and EC smoking by not diminishing acutely MSNA while maintaining high hemodynamic load and heart rate [4]. Thus, clinical research in these higher risk cardiovascular states for the impact of TC and EC smoking on sympathetic tone will provide additional pathophysiological explanations for their augmented overall risk. Especially in the elderly, it was shown that heart rate is less increased after smoking compared to younger subjects and, also, the MSNA-related sympathetic activity is not suppressed, rendering smoking even more hazardous [17]. Our study population consisted of males with a mean age of 34 years and, thus, older than the participants in another previous study in which the sympathetic response to TC and EC smoking was more pronounced [16]. This age gap could partially explain the differences in MSNA and SSNA reaction to acute smoking exposure between our study and the previous one [16]. Finally, our population consisted of smokers in contrast to the recently published work by Gonzalez J et al. [23] in which only non-smokers participated. It is possible that TC and EC smoking might have a diverse effect on autonomic regulation and hemodynamic modulation according to the smoking status of the studied subjects [16,17,23] 

Cardiac mortality associated with smoking is non-linear with the risk being binary, meaning that the consumption of 1–3 cigarettes per day correlates to the risk induced by 20 cigarettes per day [28]. On these lines, with the acute exposure to TC and EC smoking, the unfavorable changes in mean arterial pressure and heart rate along with MSNA and SSNA are observed with the first cigarette and maintained after the second one, and, in the case of EC, at 5 min the impact is comparable to that of 30 min. Given that experienced EC smokers can increase their nicotine absorption significantly by puff duration compared to naïve subjects [33] such as those in our study, it is possible that the unfavorable effects of vaping are underestimated. These results suggest that exposure to low levels of nicotine is adequate to elicit important systemic changes in humans that are then enhanced and maintained by the continuation of TC or EC smoking. Specific for our study, the alterations of sympathetic drive by TC and EC smoking were evident in the already increased sympathetic activation that is present in habitual TC smokers [18]. 

The study results are limited by the inclusion of only males; however, there are gender differences to blood pressure effects of smoking, and this baroreflex diverse reaction to acute TC and EC exposure could influence the sympathetic response [4]. The lack of active nicotine and cotinine estimation along with no spontaneous cardiovagal baroreflex sensitivity calculation limits our findings, along with the fact that smoking with a drinking straw is still different than the physique of the cigarette and subjects were not blinded. Moreover, no repeated measurements for hemodynamic and sympathetic parameters on each participant were made. There was also no sham EC testing as well as no EC without nicotine as in a previous work [23]. Finally, the effect of TC and EC smoking on sympathetic drive, blood pressure and heart rate after the acute phase were not assessed. 

## 5. Conclusions

This is the first study showing that inhaling ECs acutely increases mean arterial pressure and heart rate, decreases MSNA and augments SSNA in healthy smokers. These findings provide novel insights into the unfavorable impact of vaping on the cardiovascular system and support further research for the overall effects of EC.

## Figures and Tables

**Table 1:** Effects of TC and EC smoking on hemodynamic parameters and on sympathetic activity.

Parameter	Baseline	TC Phase 1	TC Phase 2	*p*	Baseline	EC Phase 1	EC Phase 2	*p*
**Systolic BP (mmHg)**	117 ± 5	126 ± 6	129 ± 5	<0.001	119 ± 5	127 ± 6	133 ± 5	<0.001
**Diastolic BP (mmHg)**	67 ± 3	71 ± 3	73 ± 3	<0.001	68 ± 4	72 ± 4	75 ± 5	<0.001
**Heart rate (bpm)**	64 ± 5	72 ± 6	76 ± 5	<0.001	66 ± 6	71 ± 6	75 ± 8	<0.001
**MAP (mmHg)**	84 ± 3	90 ± 3	92 ± 3	<0.001	84 ± 3	90 ± 3	94 ± 4	<0.001
**MSNA (bursts/min)**	23 ± 3	17 ± 3	17 ± 4	<0.001	24 ± 3	16 ± 4	16 ± 3	<0.001
**SSNA (bursts/min)**	13 ± 2	22 ± 3	23 ± 3	<0.001	13 ± 2	20 ± 2	22 ± 2	<0.001
**Plasma NE (pg/mL)**	168 ± 19	170 ± 22	182 ± 27	0.002	173 ± 22	185 ± 17	231 ± 30	0.003

BP, blood pressure; EC, electronic cigarette; MAP, mean arterial pressure; MSNA, muscle sympathetic nerve activity; NE, norepinephrine; SSNA, skin sympathetic nerve activity; TC, tobacco cigarette. *p*-values for differences within a session were obtained by post hoc tests (planned contrasts). Results are expressed as mean ± standard deviation (SD). All tests were two-sided and *p* < 0.002 was considered significant after Bonferroni adjustment for multiple comparisons.

**Table 2:** Effects of sham smoking on hemodynamic parameters and on sympathetic activity.

Parameter	Baseline	Sham Smoking Phase 1	Sham Smoking Phase 2	*p*
**Systolic BP (mmHg)**	114 ± 5	113 ± 4	112 ± 5	0.135
**Diastolic BP (mmHg)**	65 ± 3	66 ± 3	64 ± 3	0.094
**Heart rate (bpm)**	63 ± 4	64 ± 5	64 ± 5	0.041
**MAP (mmHg)**	84 ± 3	82 ± 3	80 ± 3	0.001
**MSNA (bursts/min)**	24 ± 2	23 ± 2	23 ± 3	0.023
**SSNA (bursts/min)**	15 ± 2	14 ± 2	15 ± 3	0.076

BP, blood pressure; MAP, mean arterial pressure; MSNA, muscle sympathetic nerve activity; SSNA, skin sympathetic nerve activity. *p*-values for differences within a session were obtained by post hoc tests (planned contrasts). Results are expressed as mean ± standard deviation (SD). All tests were two-sided and *p* < 0.002 was considered significant after Bonferroni adjustment for multiple comparisons.
